# The sick child in early modern England, 1580–1720

**DOI:** 10.1016/j.endeavour.2014.04.001

**Published:** 2014-06

**Authors:** Hannah Newton

**Affiliations:** University of Cambridge, History and Philosophy of Science, Free School Lane, Cambridge CB2 3RH, United Kingdom

## Introduction

One morning in 1630, fourteen-year-old Richard Wilmore from Stratford vomited ‘black Worms, about an inch and a half long, with six feet, and little red heads’. After vomiting, he ‘was almost dead, but a little time after he revived’. The next day, the boy's father went to see a doctor called John Hall, ‘earnestly desiring’ his advice. He brought with him some of the worms ‘wrapped in Paper’, which, upon examination, ‘crept like Earwigs, and were very like, save in colour’. Richard was so ‘cruelly afflicted’ that ‘he was ready to tear himself in pieces’. Dr Hall administered a medicine which made the boy vomit seven times, and bring up ‘six Worms’, the like of which the doctor had ‘never beheld or read of’ before. Dr Hall noted with satisfaction that this treatment ‘delivered’ Richard from his infestation, so that when ‘I met him two years later’, he ‘gave me thanks… [and] told me he had never been troubled with it since’.^1^

**Figure 1:**
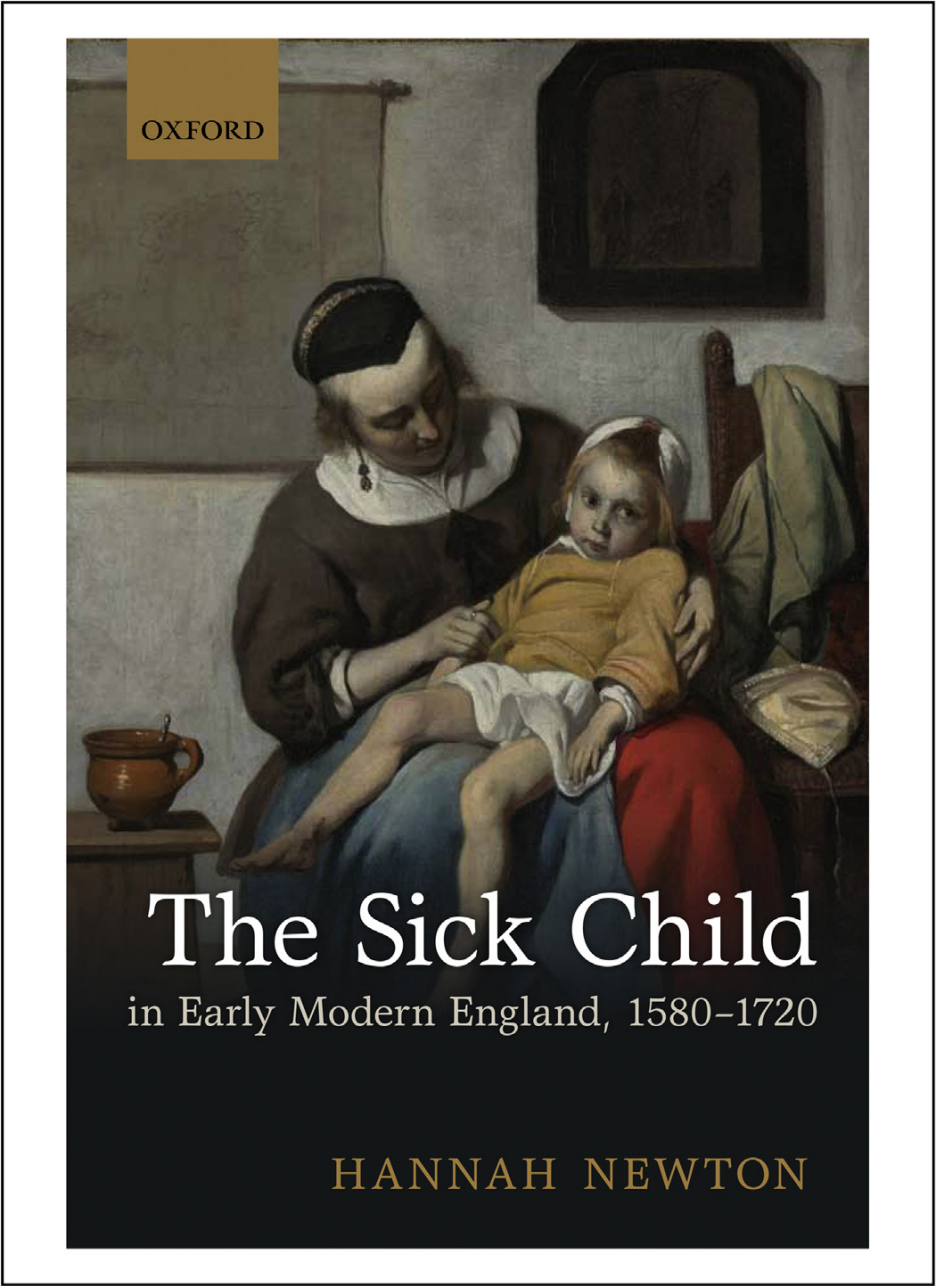
*Jacket illustration*: The Sick Child (c. 1660–1665) by Gabriel Metsu (1629–1667).

I encountered this bizarre case in the published medical notebook of the eminent Stratford physician John Hall, who happens also to have been Shakespeare's son-in-law. It sparks a host of questions about childhood illness in early modern England. Were children's medicines the same as those of adults? What role did parents play in the care of ill offspring? How did young people like Richard respond emotionally to illness and suffering? These are some of the questions addressed in my book, *The Sick Child in Early Modern England*.^2^ Taking the triple perspectives of doctors, parents, and children, the book investigates the perception, treatment, and experience of childhood illness in England between approximately 1580 and 1720. At this time, almost a third of young people died before the age of fifteen, and yet comparatively little research has been undertaken on this subject.^3^ Drawing on sources such as doctors’ casebooks, medical texts, personal documents, and eulogies, the book overturns three major historical myths [Figure 1].

## Myth 1: children were miniature adults

The first myth, is that for much of the early modern period, children were regarded as miniature adults. This idea is most famously associated with the French scholar, Philippe Ariès, whose book *Centuries of Childhood* (1962), argues that the concept of childhood did not exist in pre-modern societies, as evidenced by the tendency of artists to depict children in adult dress.^4^ This view has largely fallen out of favour amongst historians of childhood, but in the context of medical history, it lives on, with scholars continuing to assert that until as late as the nineteenth century, doctors neither recognised ‘the physiological differences’ between children and adults, nor ‘acknowledged the need for… treatment designed specifically for children's unique physiology’.^5^

A foray into the medical sources of early modern England shows that this was not the case: children's bodies, diseases, and treatments, were distinguished fundamentally from those of adults. The physiological uniqueness of children resided in their ‘humours’. Rooted in Hippocratic and Galenic medical traditions, it was believed that all living creatures were made up of four fluids called humours – blood, choler, melancholy, and phlegm.^6^ Each humour was characterised by its particular temperature and moisture content, and it was the combination of these qualities that enabled the body and mind to function. The balance of humours was believed to alter over the course of the life-cycle. The physician J.S. explained in 1664, ‘The Life of Man consists in Heat and Moisture, the Heat consumes by degrees the Moisture, whereby necessarily follow several Changes of the Temperament, which are called Ages’.^7^ The life-cycle was divided into four ages: childhood (from birth to fourteen); youth (from fifteen to the mid-twenties); adulthood (from mid-twenties to the mid-fifties); and old or ‘decrepit’ age (from mid-fifties until death). At birth, living beings were warm; the temperature then increased until the end of youth, after which point it steadily lessened. Moisture, by contrast, was greatest at birth, and from that moment onwards was in decline. Death occurred when all the moisture and heat had been depleted. Ageing was thus a cooling and drying process.

According to this theory, children were more warm and moist than other ages, abounding in the humour blood. This great humidity made their bodies weaker and softer than those of adults. The royal physician Walter Harris wrote in his 1693 medical text, ‘children's’ [flesh], bones and cartilages, are like soft Wax, curdled or gathered butter… or sammed cheese’, whereas in old men, they are ‘dry and wither’d’.^8^ Children's humidity also affected their minds – as the Oxford academic Henry Cuffe stated in 1607, ‘in their infancie’ children have ‘no actuall evident use of their reason’, because their brains are ‘drowned and drunk with moisture and humours’.^9^

**Figure 2:**
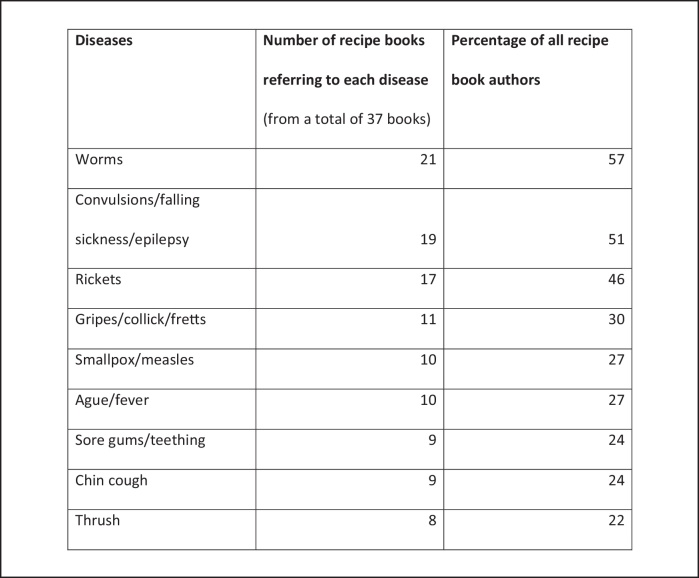
A table to show the most commonly cited children's diseases in recipe books.

Children also differed from adults in their disease vulnerability. Early modern paediatric treatises usually list between thirty and forty-five diseases, which include conditions as diverse as ‘nightmares’, ‘pissing the bed’, and ‘breeding of teeth’ (teething). An analysis of domestic recipe books – collections of manuscript medical recipes for use in the home – indicate that laypeople also recognised children's susceptibility to a particular range of diseases. The most commonly cited diseases in these recipe collections are listed in Figure 2.

What were the causes of these diseases, and were they specific to children? The overarching cause of illness was sin: God sent disease as a punishment for human transgressions. God's method for bringing disease into fruition was the imbalance or corruption of the bodily humours, a theory of causation which was the same for all ages. However, crucially, many of the factors that precipitated the humoral imbalance *were* distinct to children. The most important, was the child's humidity. As explained by J.S., ‘every Age hath a peculiar temper, and so a similtude with some Diseases’.^10^ Since diseases were caused by particular combinations of humours, individuals were pre-disposed to those diseases which shared their own natural constitution. Thinking back to Richard Wilmore, contemporaries would have probably attributed his worm infestation to the fact that, as one physician put it, ‘Worms… are generated by… [children's] hot and moist constitution, which is very apt to produce Worms; and the sweet things which Children eat, and are delighted with’.^11^ Another child-specific cause was the corrupt menstrual blood of mothers, which was thought to seep into the fetus’ body during gestation, and cause many ailments throughout childhood. Speaking of smallpox in 1700, the medical author Robert Johnson wrote, ‘[it is caused by] an ill quality or impurity of the Mothers bloud, with which the Child was nourish’d in the Womb… by which nature is inraged and provoked to cast forth the impurity’ through the pustules of the smallpox.^12^ Any disease characterised by some sort of mark on the skin was usually blamed on the menstrual blood.

**Figure 3:**
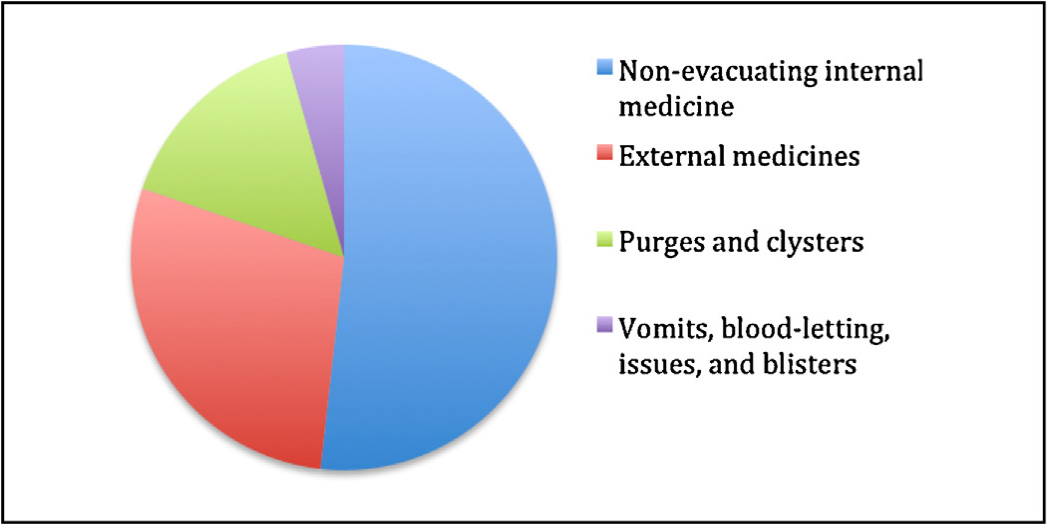
A pie chart to represent the popularity of various treatments in recipe books.

Turning from disease to treatment, children's medicines differed from those of adults. ‘A special regard’, declared the English physician John Pechey (c. 1655–1716), ‘is to be had to the Methods and Medicines, for Children by reason of the weakness of their bodies, cannot undergo severe methods or strong Medicines’.^13^ Instead of using the usual remedies of the day – vomits, purges, and bloodletting – children were to be treated with milder medicines, such as topical ointments and baths, and non-evacuating internal medicines. Of the 482 medicines for children that were listed in the collections of manuscript medical recipes that I analysed in the Wellcome Library and British Library, less than 4 percent were for vomits and bloodletting, and only about 15 percent were for purges and enemas. These percentages are represented in Figure 3. Evacuative treatments were to be avoided because they were, in the words of the Fellow of the Royal College of Physicians, Francis Glisson, ‘unpleasing, ful of pain and molestation to Children’.^14^ Of course, there were occasions when these treatments were used – as we saw with Richard Wilmore, older children were more likely to be given vomits and purges than infants, and when the child was gravely ill, practitioners were sometimes prepared to risk administering a more aggressive remedy on the grounds that it might save the child's life.

**Figure 4:**
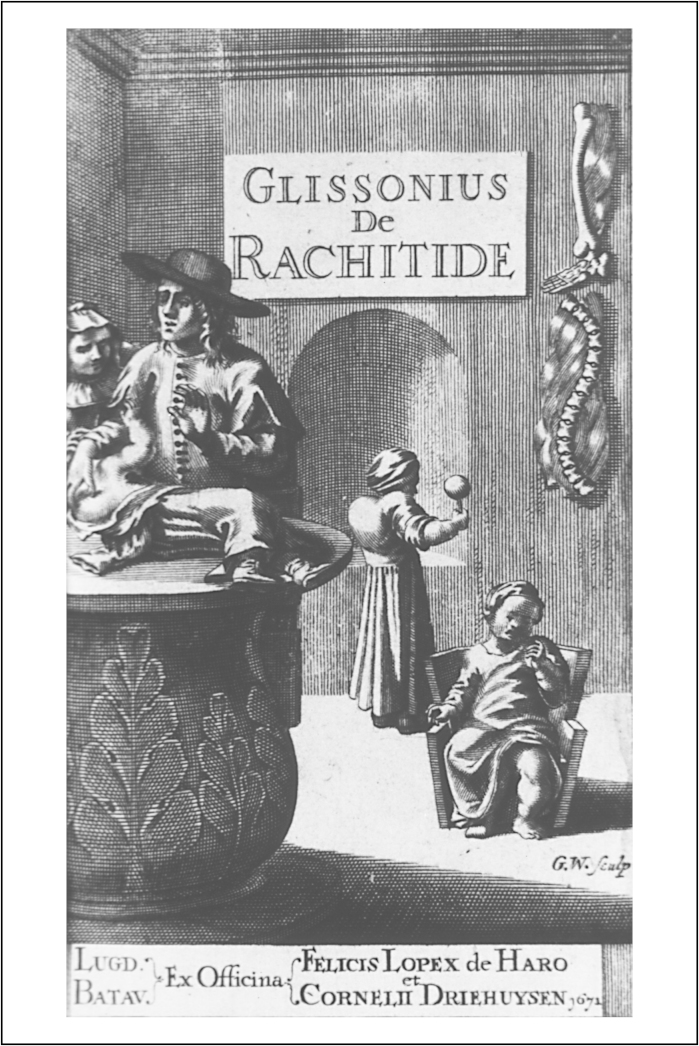
The frontispiece of Francis Glisson, *De rachitide, sive morbo puerili, tractatus* (Leiden, 1671); image supplied by Light, Incorporated. A child is holding a rattle – a method of distraction for children in pain.

As well as giving children different treatments, it was considered necessary to adapt the medicines in various ways. Doses were lessened for children, and the more powerful ingredients were often omitted. The Brumwich family recipe book, dated 1625, stated that ‘a man’ must ‘Take 11 drops’ of the ‘spirit of Vitterell’, while ‘a child of 10 years old not above 6 or 7 drops’, ‘a child of 5 years old not above 4 drops’, and finally ‘a child of 3 years old not above 2 drops’.^15^ Mary Poppins’ technique of ‘adding a spoonful of sugar’ to disguise bitter tastes was another common practice. Glisson noted that he sought to make his medicines ‘grateful & pleasing to the sick Child, & such as trouble not its Pallate’.^16^ This was because the ‘teats’ of the tongue, the regions responsible for taste, functioned most acutely in childhood. Doctors also paid special attention to pain-relief in children, commanding that their priority was to ‘First abate Pain’.^17^ This preoccupation stemmed from the belief that pain was particularly damaging to children. To this end, poppies, mallows, and lettuce were added to medicines, although opiates were rarely used internally. Distraction was also employed: the French midwifery writer François Mauriceau suggested in 1710 midwifery manual, that infants suffering from painful teething should be given ‘a Silver Coral, furnish’d with small Bells, to divert the Child from the Pain it then feels’.^18^ Figure 4 depicts a child with rickets playing with a rattle; this may have been intended as a method of distraction.

In short, rather than regarding children as miniature adults, doctors and laypeople depicted this age-group as medically distinct. The term I have coined to refer explicitly to this notion, is ‘children's physic’. Through this research, I hope to highlight the importance of age more generally in early modern times: historians have tended to favour gender over age as a category of analysis, and therefore my book seeks to raise the profile of this other, crucial variable.^19^

## Myth two: parents did not love their children

We now reach the second myth: that high rates of death in the early modern period discouraged parents from investing too much affection in their children.^20^ Although this interpretation has been challenged in recent years, scholars have continued to view fathers in this way – they are depicted as unemotional, aloof figures, who spent little time with their children, and rarely showed much grief at their deaths.^21^ It is said that the ideology of patriarchy would have rendered the physical care of sick children a ‘questionable’ task for men.^22^

Contemporary sources throw doubt on these assumptions. During illness, mothers *and* fathers tended their children with devoted care, bestowing earnest prayers, and nursing their offspring day and night. In 1679, the East Anglian clergyman Isaac Archer recorded that he ‘sate by’ his six-year-old daughter Frances all night, and ‘wrestled with God with much earnestnes for the child's life, so as I never was in such anguish before’.^23^ Fathers themselves do not seem to have regarded these tasks as feminine. On the contrary, when the child was suffering from a particularly dangerous illness, they sometimes hinted that nursing required courage and rationality, attributes associated with masculinity. This was implied by Isaac Archer, who recorded that, ‘My wife could not helpe her [daughter Frances] through griefe’.^24^ This round-the-clock care was exhausting to parents, sometimes affecting their health. In 1650, the Oxfordshire physician, Thomas Willis attributed the death of his forty-year-old patient to her having ‘spent many sleepless nights nursing a sick child’.^25^

Parents also spent much time preparing and administering medicines. To give one example, the Trumbell family's recipe for ‘plague water’ required parents to,

Take Egremony Rue Wormwood, sollendin, Angelica Sage, Tormentil…Scabius, Baume, Mugwort, Pimpernell, Spermint, Scordium or Scordus, Cardus, Dragons, Fetherfew, Galiga, Rosasolis, Lilly of the vally, Marygold flowers, Barage flowers, Cowslip flowers, Pancy flowers, of each of these a quarter of a pound. Fenell seeds, Coriander seeds and Anniseeds, Cardimum seeds of each an ounce, half a pound of Rosmary Ledoary or Zedaree halfe an ounce Scorzonera a handfull, shred the herbs small… putt them into an earthen pott well glazed, then put into the pott 3 gallons of Sack & then cover it & past it up very close let it stand 8 or 9 dayes, then put it into the still & add to it 2 ounces of fine Methridate 3 ounces of Venice Treacle, of Cinnamon, Cloves & Nutmegs of each halfe an ounce & still it gently.^26^

The processes involved in concocting medicines could be physically arduous, as well as tedious, to carry out.^27^ Love for children is also demonstrated by the fact that families were prepared to spend great sums of money on medical treatment. When Lady Elizabeth Bradshaigh's two grandsons caught smallpox in 1687, she complained that the total cost incurred to pay for ‘the doctors’, nurses, and the ‘[a]pothecary's bill’, amounted to ‘near twenty pounds’. She added, ‘to save the pretty boys’ lives, I am content to do anything’.^28^ To put this in context, the average wage of a skilled craftsman in the late seventeenth-century was £54 a year.^29^

**Figure 5:**
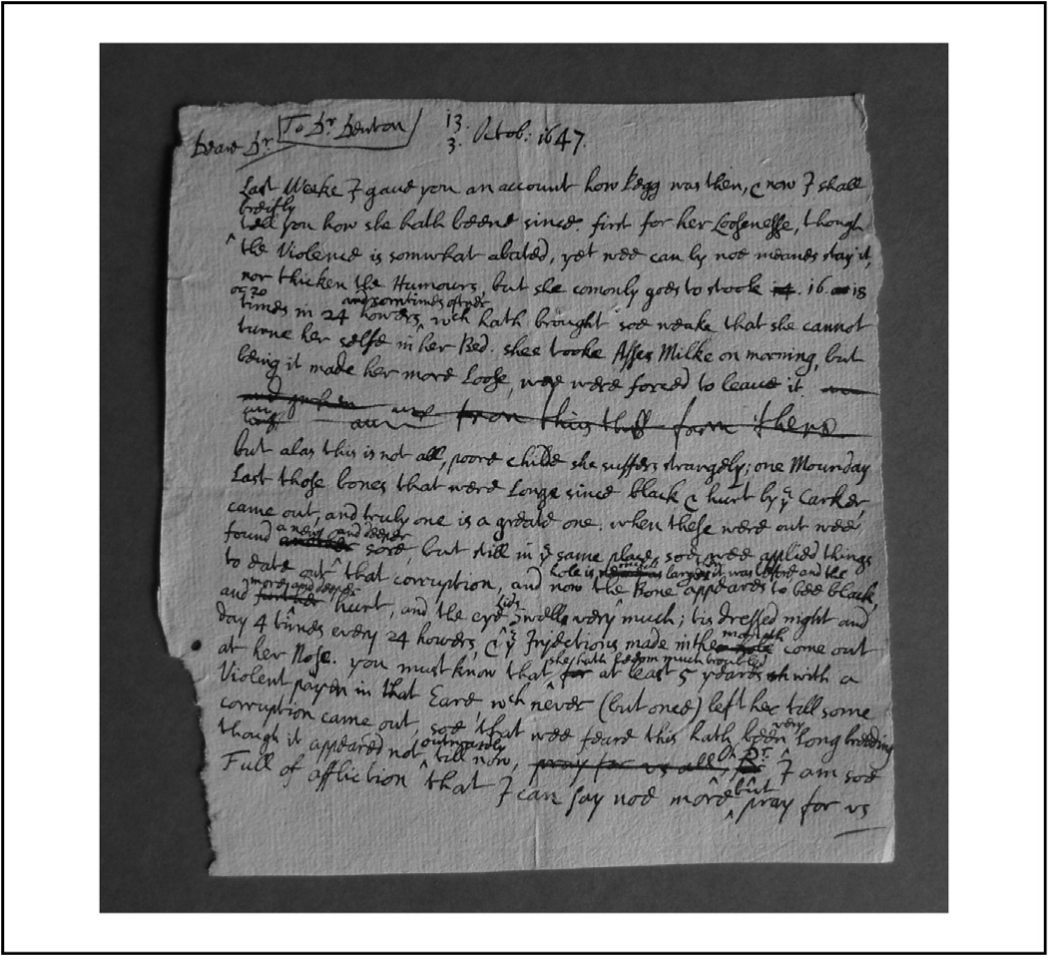
A draft letter by Ralph Verney about his ill daughter Pegg, 13/3 October 1647; by kind permission of Sir Edmund Verney, and with the assistant of the archivist, Mrs Sue Baxter.

The most poignant evidence of parental affection is their expression of grief. Grief was defined as ‘*a violent* passion *of the Soule, entertained by some sensible discontent*… *a torment of the mind*’*.*^30^ In this period, the emotions were called ‘passions’: they were ‘motions of soul’, which meant physical movements, instigated by the middle part of the soul for the preservation of the human being.^31^ The passions were depicted as powerful liquids, linked to the bodily humours, which surged through the body bringing physical effects. Grief began before the child had actually died, and was sparked by observing the child's suffering. In 1647, the Yorkshire gentleman Ralph Verney wrote to his uncle Dr Denton, about the cancerous ulcer and diarrhoea of his eight-year-old daughter Pegg: he lamented, ‘Poore childe you doe not know what miserie she hath endured’: ‘she comonly goes to stoole 16, 18, or 20 times in 24 howors…w[h]ich hath brought [her] soe weake that she cannot turne her selfe in her Bed’. He concluded his letter, ‘oh Dr I am so full of affliction that I can say noe more but pray for us’.^32^ This letter is presented in Figure 5.

Witnessing the actual moment of death caused unspeakable distress. After several months of illness in 1665, twelve-year-old Caleb Vernon from London finally announced, ‘Now I think I shall die’. Seeing his father ‘gush out into tears’, Caleb cried ‘Father do not weep, but pray for me[:] I long to be with God’. He began to grow breathless, ‘as if choaked with plegm’, and his, who was ‘in great care for him’, father ran downstairs to fetch some medicines ‘for his relief’. Returning quickly, he saw his son ‘thrusting, first, his finger, and then his whole hand in to his mouth’ to clear his throat. Hearing his father coming, Caleb gasped, ‘O Father, what shall I do?’, and then ‘immediately lay back’, uttered ‘God, God’, and died.^33^ This disturbing image haunted Caleb's father for the rest of his life.

**Figure 6:**
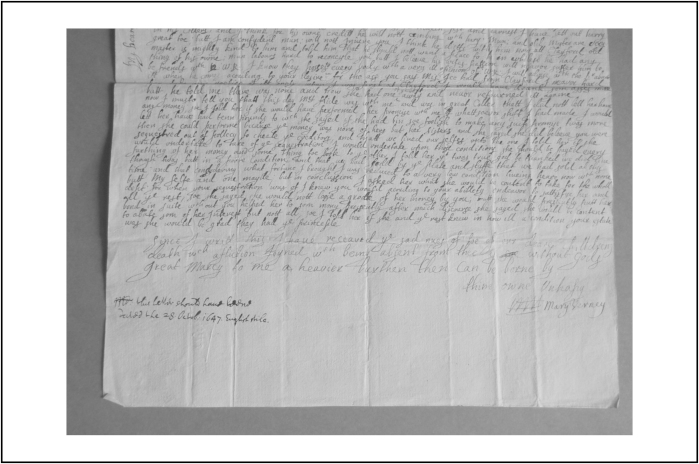
A letter from Mary Verney to her husband Ralph Verney, 28 October 1647; by permission of Sir Edmund Verney, and with the assistance of the archivist, Mrs Sue Baxter.

The next stage of grief occurred in the minutes, hours, and days after the child's death, and was labelled ‘distraction’ by contemporaries. In the same week that Ralph Verney's daughter Pegg died, her baby brother Ralph also passed away. Their mother, Mary, was in the middle of writing a letter to her husband when she found out about their deaths. The surviving letter, Figure 6, provides an exceptionally rare, and heartrending insight into the experience of early grief. She breaks off half way through with the words, ‘Since I writt this, I have received the sad nues of toe of our deare chilldrens death, which affliction Joyned with being absent from thee is without Gods great Marcy to me a heavier burthen then can be borne by thine M’^34^. Mary's handwriting, rather than her words, express her extreme sorrow, for it changes from a neat italic hand to a larger scrawl. A member of Mary's household reported that the tragic news ‘did much afflict and distract her, soe that she spake idly for two nights and sometimes did not know her frends’.^35^

‘Distraction’ referred to a grief that verged on madness: it was characterised by periods of delirium and weeping.^36^ Early modern philosophers believed that ‘Sighing, groaning, and weeping’ had ‘a deeply therapeutic function’^37^: the French philosopher Nicholas Coeffeteau explained,

[W]ee finde many times in our bitterest griefes, that teares diminish our paine, and mollifie our miseries… when wee powre forth teares, we cast out that which afflicts us, & emptying the humor which oppresseth us, and smothers us within, by this meanes we free our selves from a heavy burthen which lay upon our hearts.^38^

Tears were thus associated both with the humours and with the passions, providing a link between the spheres of body and mind.^39^

In the years that followed a child's death, parental sorrow often persisted, though it may have lost some of its sharpness. Certain situations could rekindle the painful remembrances. The Verney parents were asked if they could look after a friend's little girl one day: Mary replied, ‘Since itt has pleased god to take away.. my deare gerle’ Pegg, ‘I cannot have patience to lett any Bodies elces [daughter] dwell with me[,] for the sight of them in my owne howse would butt make my wound dayly bleed afresh’.^40^ Her husband agreed, saying, ‘It would renew your griefe, and breake my hart, for I confesse noe creature knew how much you loved that poore childe’. As well as revealing the mutual love and understanding between this couple, the images of the bleeding and broken hearts confirm the impression that the passions were thought to be tangible liquids which seeped from the heart.^41^ In short, all the evidence points to the intensity of love between parents and their children. By demonstrating the crossover of men and women's emotional and practical responses to child sickness and death, my book suggests that we revise our picture of gender roles in the early modern period.

## Myth 3: it is impossible to access the child's experience

The third myth is that it is impossible to investigate the experience of childhood in the early modern period because children rarely left written records.^42^ As Peter Stearns has stated, the ‘granddaddy issue’ faced by historians of childhood is the ‘virtually unprecedented’ problem of ‘getting information from children themselves, as opposed to adult perspectives’.^43^

But, there is one context in which children's voices can be heard: illness. Acutely aware of the likelihood of death, parents and relatives recorded the thoughts, words, and actions of their offspring in detail, conscious that these might soon be cherished as last memories. The resulting evidence provides rare and intimate insights into the lives, illnesses, and deaths of early modern children. At bedtime in 1625, three-year-old Elizabeth Wallington from London, ‘then being merry’, said to her father, ‘Father I goe abroode tomorrow and bye you a plomee pie’. The reason this everyday sentence was recorded by Elizabeth's father was that, ‘These were the last words that I did heere my sweete child speeke’, for a few hours later, ‘the very panges of death seassed upon her… [which] were very grievous unto us the beholders’, and she died at four o’clock in the morning.^44^ Drawing on evidence of this kind, my book views sickness through the child's eyes: it explores the emotional, spiritual, physical, and social dimensions of illness, pain, and death.

Children's emotional responses to pain were sometimes recorded in parents’ diaries. In 1650, the Essex puritan clergyman Ralph Josselin lamented that his eight-year-old daughter Mary, who was dying from worms, was ‘heavy, and joylesse’, pointing to her tummy, and crying out, ‘poore I, poore I’.^45^ These doleful responses were put down to the sympathy between the body and soul. The French philosopher Jean-Francois Senault (c. 1601–1672) declared, ‘when the body is assaulted… [with] the rage of Sickness, [the soul] is constrained to sigh with it… the Cords which fasten them together make their miseries common’.^46^ Hence it was considered inevitable that physical pain would provoke emotional distress.

Young people's attitudes to medical treatments frequently emerge in the sources. Following several months of wearing a brace to correct his crooked back in the 1650s, fifteen-year-old Edmund Verney told his father, ‘I may truly say the cure is almost perfected’; he added with glee, ‘I am almost as tall, if not taller than my cousin Spenser’. This cure came at a price, however – in a previous letter, Edmund had complained that, ‘[A]fter first wearing the brace I found that I was in great pain… when all nine screws are in place [it causes] the continual heat of my body [which] is trapped in me always’.^47^ This source is all the more valuable because it was written by the boy himself, as opposed to an adult observer, though of course, we should remember that children's writings were often corrected or edited by adults.

Children's responses to other aspects of their care can sometimes be glimpsed. In 1661, the father of thirteen-year-old James Barrow from London ‘set apart’ three entire days for the seeking of ‘the Lord in behalf of my Child’.^48^ This boy found the lengthy prayer tedious. On the third day of prayer, he cried out: ‘What three dayes! two dayes was enough…What, nothing but pray! what, all pray! all mad! will you kill your selves with praying? Three dayes is too much’. Incidentally, James’ outburst was interpreted as the voice of the Devil, but it nevertheless provides an insight into this child's experience.

While sickness was undoubtedly distressing in many ways, the picture is not entirely bleak. Often, children seem to have derived comfort from the tender care they were shown by their families. The Yorkshire Quaker Thomas Camm described how his eight-year-old daughter Sarah lay in his arms during her illness in 1682, and told him, ‘*Oh! my dear Father, thou hast been very tender and carefull over me, and hast taken great pains with me in my Sickness*’*.*^49^ This father had kept notes as he sat by his daughter's bedside, recording verbatim all her ‘Memorable and weighty sayings’. Parents went to great lengths to comfort and cheer up their sick children. Caleb Vernon, who we encountered earlier, was ‘very much eased’ and ‘pleased’ upon hearing his father's ‘expressions of affection’ for him during his illness. When he found himself ‘inclining to melancholy’, he asked his parents to bring him ‘a young Lamb, Pigeon, [or] Rabbit’, in order to divert him from his pains, and provide ‘pretty company for me’. Remarkably, his parents granted his request, deciding that a pet squirrel would be best, because ‘it might easily be procured’.^50^ Perhaps sickness could be an empowering experience for children, as they were permitted to make great demands on their parents.

What did children think about death? It is often assumed that such questions are beyond the scope of historical enquiry, due to scant evidence.^51^ But, parents’ diaries and letters are replete with descriptions of children's emotional responses. One of the key tasks of carers was to help the sick prepare spiritually for death – this meant making sure they were aware they were dying, and encouraging them to perform acts of piety, such as repentance. Parents were thus keen to record their attempts at carrying out this duty, together with the child's response. ‘My dear, Are you so ill that you think you shall die?’, enquired the mother of thirteen-year-old Margaret Andrews three hours before her death in 1680.^52^ While this practice of questioning children on such a foreboding a subject might seem cruel today, in this period it was considered quite the opposite – ultimately, the aim was to help the child reach a state of confidence and happiness about death.

Some children worried about the practical problems of death and salvation. Six-year-old Joseph Scholding from Suffolk, ‘one Morning as he lay in his Bed very ill’, said to his mother, ‘Mother… I am thinking how my Soul shall get to Heaven when I die; my Legs cannot carry it, [because] the Worms shall eat them.’^53^ Children also expressed anxiety about leaving their parents. In the 1670s, six-year-old Jason Whitrow, living in Covent Garden, took his mother ‘by the hand, and said, Mother, I shall dye, oh that you might dye with me, that we might both go to the Lord together’.^54^ It is in this context that children's love for their parents is conveyed with most poignancy.

**Figure 7:**
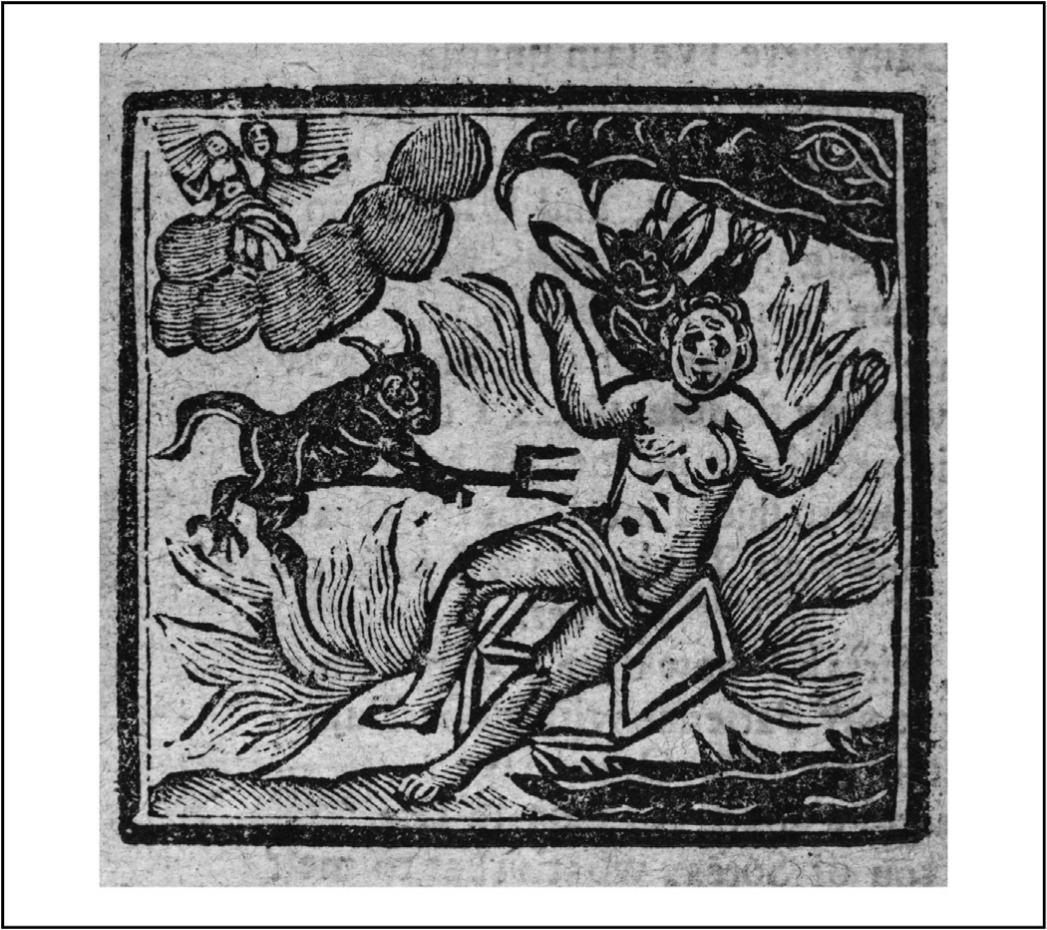
Woodcut of hell from A voice from heaven, the youth of Great Britain (1720); ^©^The British Library Board.

Infinitely more terrifying than these concerns, however, was the prospect of hell. When fifteen-year-old Joseph Taylor read ‘a little Book’, which gave ‘a Pathetical Description of Hell’, he was ‘put into sore Amazement and very great Terrour’. He sat ‘groaning in the dark’, crying ‘O!… How shall I bear the tormenting Flames of Hell for ever and ever!’^55^ Joseph's terrifying vision may have been inspired by the book, *A voice from heaven, [to] the youth of Great Britain*, published in 1690. The author warned, ‘Consider that you may perish as young as you are; there are small Chips, as well as great Logs in the Fire of Hell… The Child that will tell a Lye, must one Day roar in Hell, for a Drop of Water to cool his Tongue!’^56^ These books also contain vivid pictures of the damned in hell, such as Figure 7.

Despite the widespread awareness of hell, it seems that on the whole, children were convinced that their destiny was heaven. This prospect helped mitigate children's anxieties, enabling them to respond to death with a degree of positive resignation, and sometimes, occasionally joy. In 1644, eight-year-old Tabitha Alder from Kent had ‘a longing to be with’ God, declaring ‘in a kind of extasie of joy’, ‘[I] shall be with Jesus… and I shall live with him for ever!’^57^ These blissful feelings served to distract children from their physical pains, making suffering more bearable. While this joyful response from such a young child may seem scarcely credible to us, if we consider early modern attitudes to childhood, it begins to appear more plausible. At this time, children enjoyed a ‘special religious status’: they were thought to be particularly beloved by God, and capable of ‘startling divine insight’.^58^ The young had committed fewer sins than adults, despite the inheritance of original sin. These ideas were rooted in the Biblical passage, Matthew 18, verses 3–5:

And Jesus called a little child unto him, and set him in the midst of them, and said, Verily I say unto you, Except you be converted, and become as little children, you shall not enter into the kingdom of heaven. Whosever therefore shall humble himself as this little child, the same is greatest in the kingdom of heaven. And whoso shall receive one such little child in my name receiveth me.^59^

Given this cultural backdrop, and the intense religious conditioning of children from an early age, it is quite conceivable that some children would have been able to attain a sophisticated knowledge of Christian doctrines, which may then have shaped their emotional responses to death.

There were a number of powerful reasons why the prospect of death may have been welcomed by children. Firstly, children seem to have had especially vivid imaginations of heaven. When ten-year-old Christian Karr was seriously ill in 1702, she told her family, ‘O I think I see Heaven, I think I see Heaven, That is a glorious sight… the Walls and the streets of that City are like burning Gold. And I think I see all the Saints, arrayed in Whyte there’.^60^ Secondly, children wished to escape their suffering. Having wasted away over the course of several months Caleb Vernon's bones had become ‘so sharp as if they would pierce his skin, having no flesh to interpose in any part’. Overcome with ‘weariness and impatience’ from pains, he cried, ‘*It is better for me now to dye than to live*’.^61^ Given the limited availability of pain relief at this time, Caleb's response seems only natural. Thirdly, children looked forward to being reunited with their whole family in heaven. In 1620, ten-year-old Cecilia D’Ewes from Dorset contracted smallpox; her mother had died a short time previously, and therefore, she appeared not to mind dying, but instead cried with relief, ‘I will go to my mother, I will see her; I shall shortly be with her’.^62^ The demographic historian Peter Laslett has calculated that 29 percent of children would have lost their fathers by the age of fifteen; although we don’t know the figures for mothers, it is likely that Cecilia's experience was not unusual.^63^

In short, illness provides a unique opportunity to glimpse the feelings of early modern children. I have argued that their experiences of sickness were profoundly ambivalent: illness was often painful, frightening, and a source of spiritual anxiety. But on the other hand, it could be a time of love, attention, and power, and occasionally spiritual joy. This mix of positive and negative experiences stemmed from the religious doctrines of salvation and providence, together with the more practical and social consequences of patienthood. The idea that illness could in some ways be experienced positively acts as an antidote to the common historiographical assumption that sickness was a purely miserable experience, owing to the backwardness of early modern medicine.^64^ The interpretation also nuances our understanding of the psychological culture of Protestantism, by demonstrating that Calvinist doctrines could be comforting, as well as corrosive, to the morale.^65^

## Conclusion

Childhood in the early modern period has often been depicted as a miserable experience, characterised by disease, physical hardship, and aloof parenting. My research on children's sickness adds to the growing view amongst historians that society at this time was rather more humane than we might think. Children were not regarded as miniature adults: they were distinguished fundamentally from other ages in their physiology and medical treatment. Abounding in moist and warm humours, they were vulnerable to a different set of diseases, and in need of medicines of a gentler nature. Parents loved their children with the same intensity that we would expect today. The sheer effort, time, and emotion devoted to sick and dying children testifies the extraordinary affection of both fathers and mothers, for their offspring. I’ve sought to show that it *is* possible to capture the experiences of the young, even though the evidence is often indirect. By investigating the subject of children's illness, the wider aim of my research is to draw together the histories of medicine, family, religion, and emotion, fields which have rarely enjoyed much interaction.

**Figure 8:**
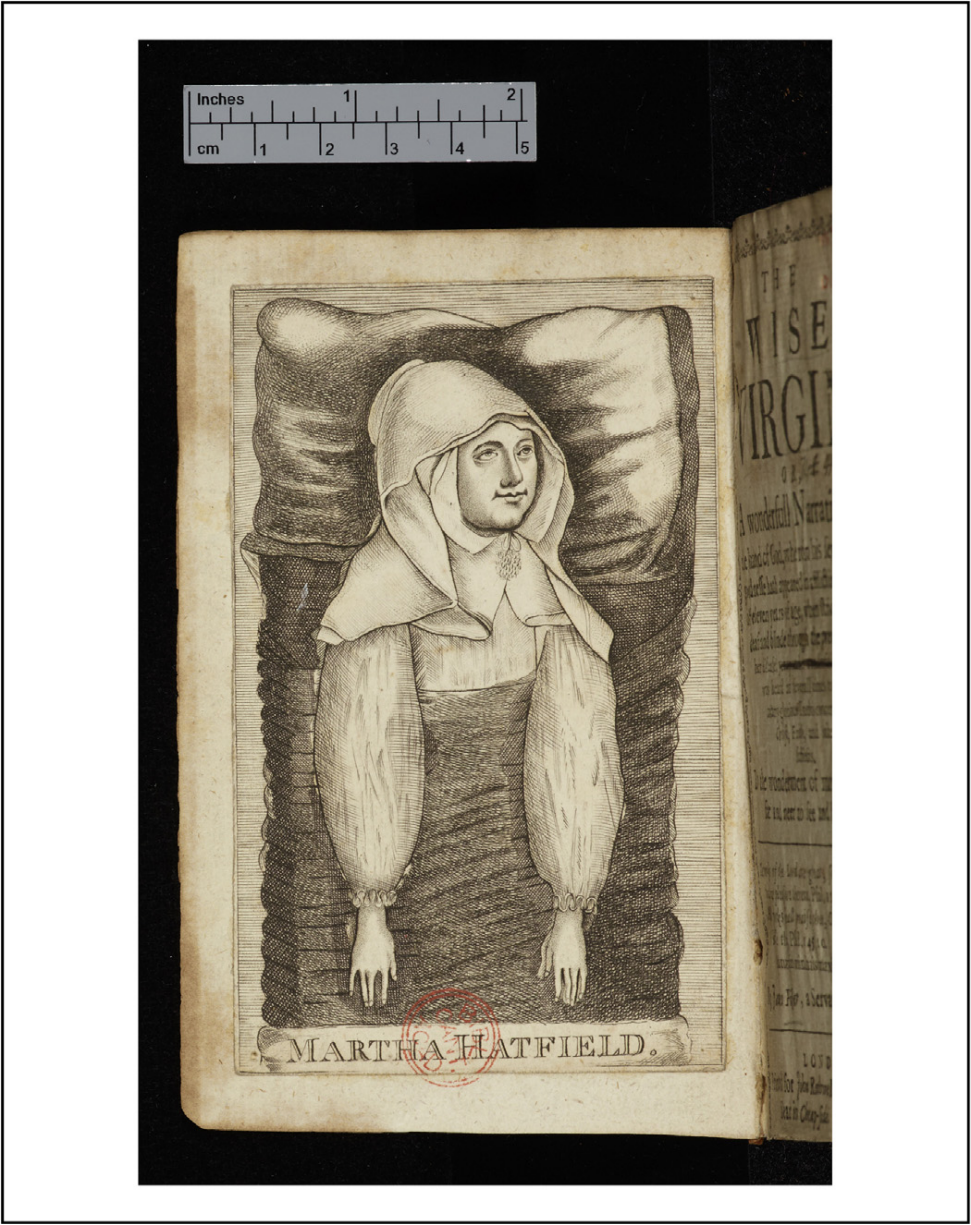
The frontispiece of James Fisher, The wise virgin, or, a wonderfull narration of the hand of God… in afflicting a childe of eleven years of age [Martha Hatfield] (London, 1653).

To end on a cheerful note, children did not always die: almost two thirds of children lived beyond the age of fifteen.^66^ Just as parents endeavoured to remember their children's last words, they were keen to record their first words upon recovery. In 1652, eleven-year-old Martha Hatfield from Yorkshire depicted in Figure 8, had been suffering from ‘spleen wind’ for nine months. One December morning, Martha suddenly felt strength returning to her limbs. She told her father, ‘It trickled down, and came into [my] thighs, knees, and ancles, like warm water’. Over the next week, Martha ‘encreased in strength… beyond all expectation’, and at last announced to her family, ‘me is pretty well, I praise God… I am neither sick, nor have any pain’. A day of thanksgiving was arranged to praise God for His ‘glorious end to this affliction’. One of the guests recalled that the sight of Martha, getting up out of bed, and ‘com[ing] forth into the Hall to meet and welcome us… was wonderfull in our eyes, so that our hearts did rejoyce with a kind of trembling’.^67^ It is this rather more cheerful topic – recovery from illness in all ages – that is the subject of my current research project.

## Figures and Tables

By permission of Rijksmuseum, Amsterdam.

## Footnotes

1 John Hall, *Select observations on English bodies* (1679), 72.

2 Hannah Newton, *The Sick Child in Early Modern England, 1580–1720* (Oxford, 2012; paperback edition 2014).

3 Edward Anthony Wrigley and Roger Schofield, *The Population History of England, 1541**–**1871: A Reconstruction* (Cambridge, 1981), 249.

4 Philippe Ariès, *Centuries of Childhood: a Social History of Family Life*, trans. Robert Baldick (1962, first publ. in French 1960).

5 A. R. Colon, *Nurturing Children: a History of Paediatrics* (London and Westport, Conn., 1999), xiv. See my book, *The Sick Child*, Ch. 2, footnotes 1–6 for further examples.

6 Galen: AD 129–199/217; Hippocrates: c. 460–370 BC.

7 J.S., *Paidon nosemata; or childrens diseases both outward and inward* (1664), 2.

8 Walter Harris, An exact enquiry into, and cure of the acute diseases of infants, trans. William Cockburn (1693), 3–4.

9 Henry Cuffe, The differences of the ages of mans life (1607), 127; J. S., Paidon nosemata, 87.

10 J.S., *Paidon nosemata*, 2–3.

11 John Pechey, A general treatise of the diseases of infants and children (1697), 119.

12 Robert Johnson, *Praxis medicinae reformata: or, the practice of physick* (1700), 137–38.

13 Pechey, *A general treatise*, 15.

14 Francis Glisson et al, *A treatise of the rickets being a disease common to children*, trans. Philip Armin (London, 1651), 317.

15 WL, MS 160, 27r (Anne Brumwich and others, ‘Booke of Receipts or medicines’, c. 1625–1700).

16 Glisson, *A treatise of the rickets*, 344.

17 Daniel Sennert, *Practical physick, or, Five distinct treatises of the most predominant diseases of these times* (1676), 263.

18 François Mauriceau, *The diseases of women with child… With fit remedies for the several indispositions of new-born babes* (1710), 343.

19 For a comprehensive overview of the literature on gender and medicine, see Wendy Churchill, *Female Patients in Early Modern Britain: Gender, Diagnosis and Treatment* (Farnham, 2012).

20 Lawrence Stone, *The Family, Sex and Marriage in England 1500**–**1800* (London, 1990, first publ. 1977).

21 For example, Michael MacDonald, *Mystical Bedlam: Madness, Anxiety and Healing in Seventeenth-Century England* (Cambridge, 1981), 84. However, recently this view has been contested by Patricia Phillippy, ‘“I Might Againe Have Been the Sepulcure”: Paternal and Maternal Mourning in Early Modern England’, in Jennifer Vaught (ed.), *Grief and Gender, 700**–**1700* (Basingstoke, 2003), 197–214, at 203; Joanne Bailey, ‘A Very Sensible Man’: Imagining Fatherhood in England, c. 1750–1830′, *History*, 95 (2010), 267–92; idem., *Parenting in England 1760–1830: Emotion, Identity, & Generation* (Oxford, 2012), 49.

22 Patricia Crawford, *Parents of Poor Children in England, 1580**–**1800* (Oxford, 2010), 122, 128–9, 243.

23 Isaac Archer, *Two East Anglian Diaries 1641–1729*, ed. Matthew J. Storey, Suffolk Record Society, vol. 36 (Woodbridge, 1994), 150.

24 Ibid., 156.

25 Thomas Willis, *Willis**'**s Oxford Casebook (1650**–**52)*, ed. Kenneth Dewhurst (Oxford, 1981), 79–80.

26 BL, Additional MS 72619, fol. 111v (‘Book of recipes for Trumbell's household’, late seventeenth century).

27 Jennifer Stine, ‘Opening Closets: The Discovery of Household Medicine in Early Modern England’ (unpubl. PhD thesis, Stanford University, 1996), iv.

28 Cited in Sir Arthur Wynne Morgan Bryant (ed.), Postman's Horn: an Anthology of the Family Letters of Later Seventeenth Century England (1946), 19.

29 This figure has been calculated from the data provided by Doreen Nagy in her book *Popular Medicine in Seventeenth-Century England* (Ohio, 1988), 21.

30 Nicholas Coeffeteau, *A table of humane passions* (London, 1621), 318–19.

31 On the passions, see Thomas Dixon, From Passions to Emotions: The Creation of a Secular Psychological Category (Cambridge, 2003); Gail Kern Paster, Katherine Rowe, and Mary Floyd-Wilson (eds), Reading the Early Modern Passions: Essays in the Cultural History of Emotion (Philadelphia, 2004); Susan James, Passion and Action: the Emotions in Seventeenth Century Philosophy (Oxford, 1997); Gail Kern Paster, Humouring the Body: Emotions and the Shakespearean Stage (Chicago, 2004).

32 BL, M.636/8, this manuscript is unfoliated (Verney papers on microfilm, 1646–50; letters dated 8 September to 13 October 1647).

33 John Vernon, *The compleat scholler; or, a relation of the life, and latter-end especially, of Caleb Vernon* (1666), 73.

34 MS 636/8, this manuscript is unfoliated (Verney papers on microfilm, 1646–50, a letter dated 28 October 1647).

35 Frances Verney (ed.), *The Verney Memoirs, 1600–1659*, vol. 1 (London, 1925, first publ. 1892), 382.

36 The Oxford English Dictionary Online provides the following definition: ‘Violent perturbation or disturbance of mind or feelings, approaching to temporary madness’.

37 Michael Schoenfeldt, ‘Aesthetic and Anaesthetics: the Art of Pain Management in Early Modern England’, in Jan Frans van Dijkhuizen and Karl A.E. Enenkel (eds), *The Sense of Suffering: Constructions of Physical Pain in Early Modern Culture*, Yearbook for Early Modern Studies, vol. 12 (Leiden and Boston, 2008), 19–38, at 34.

38 Coeffeteau, *A Table of Humane Passions*, 348–49.

39 For a history of crying, see Tom Lutz, *Crying: the natural and cultural history of tears* (New York, 1999). For religious attitudes to tears, see Thomas Dixon, ‘Enthusiasm Delineated: Weeping as a Religious Activity in Eighteenth-Century Britain’, *Litteraria Pragensia: Studies in Literature and Culture*, 22 (2012), 59–81.

40 BL, M.636, this manuscript is un-foliated (a letter from Mary Verney to Ralph Verney, dated 18th November 1647).

41 For the role of the heart in emotions, see Fay Bound Alberti (ed.), *Matters of the Heart: History, Medicine, and Emotion* (Oxford, 2010).

42 Margaret Pelling has suggested the reason scholars take the perspectives of adults when investigating childhood is that there is limited evidence available that was written by children: ‘Child Health as a Social Value in Early Modern England’, *Social History of Medicine*, 1 (1988), 135–64, at 137. There seems to be a rising interest in the child's voice: for example, Anthony Fletcher has explored children's experiences of school and home life, in *Growing up in England: The Experience of Childhood, 1600**–**1914* (London, 2008). Katie Barclay, Ciara Rawnsley, and Kimberley Reynolds are currently putting together a proposal for an edited volume on childhood death, which priorities the child's experience; the provisional title is ‘Small Graves: Death, Emotion and Childhood in the Early Modern Period’.

43 Peter Stearns, ‘Challenges in the History of Childhood’, *Journal of the History of Childhood and Youth*, 1 (2009), 35–42 at 35.

44 Guildhall Library, London, MS 204, [his pagination] 408–9 (Nehemiah Wallington, ‘A Record of the Mercies of God: or A Thankful Remembrance’).

45 Ralph Josselin, *The Diary of Ralph Josselin 1616**–**1683*, ed. Alan Macfarlane (Oxford, 1991), 201.

46 Jean-François Senault, The use of passions written in French by J.F. Senault; and put into English by Henry, Earl of Monmouth (1671), 478–82.

47 British Library, M.636/12, this manuscript is unfoliated (Verney papers on microfilm, 1646–50; a letter dated 24 March 1653; translated by Leona Archer into English from French).

48 John Barrow, The Lord's arm stretched out in an answer of prayer, or, a true relation of the wonderful deliverance of James Barrow (1664), 13–17.

49 Thomas Camm, The admirable and glorious appearance of the eternal God, in his glorious power, in and through a child of the age of betwixt eight and nine years, upon her dying bed (London, 1684), 5.

50 Vernon, *The compleat scholler*, 65.

51 There are a few exceptions – see Ralph Houlbrooke, ‘Death in Childhood: the Practice of the Good Death in James Janeway's **“**A Token for Children”’, in Anthony Fletcher and Stephen Hussey (eds), *Childhood in Question: Children, Parents and the State* (Manchester, 1999), 37–56.

52 The *life and death of Mrs Margaret Andrews* (London, 1680), 60.

53 William Bidbanck, A present for children. Being a brief, but faithful account of many remarkable and excellent things utter’d by three young children (London, 1685), 75–6.

54 Rebecca Travers, *The work of God in a dying maid [and her brother]…Susanna Whitrow* (London, 1677), 47–8.

55 Joseph Taylor, *Grace, grace: or, the exceeding riches of grace* (London, 1702), 7–8.

56 *A voice from heaven, the youth of Great* Britain (London, 1690), Image 7.

57 James Janeway, *A token for children. The second part* (London, 1673), 21–2.

58 Alexandra Walsham, ‘“Out of the Mouths of Babes and Sucklings”: Prophecy, Puritanism, and Childhood in Elizabethan Suffolk’, in Diana Wood (ed.), T*he Church and Childhood, Studies in Church History*, vol. 31 (Oxford, 1994), 285–300, at 286, 295.

59 Matthew 18, verses 3–5, King James Version of the Bible.

60 Archibald Deans, An account of the last words of Christian Karr, who dyed… in the eleventh year of her age (Edinburgh, 1702), 7.

61 Vernon, The compleat scholler, 66. This line is from Jonah 4, verse 8.

62 Simonds D’Ewes, The Autobiography and Correspondence of Sir Simonds D’Ewes, Bart., ed. J. O. Halliwell, 2 vols (London, 1845), 157.

63 Peter Laslett, ‘Parental Deprivation in the Past’, in his *Family Life and Illicit Love in Earlier Generations* (Cambridge, 1977), 162–3.

64 For these pessimistic interpretations, see MacDonald, *Mystical Bedlam*, 191; Guy Williams, *The Age of Agony: The Art of Healing, c. 1700**–**1800* (London, 1975).

65 Negative pictures of the psychological effects of Protestantism have been given by John Stachniewski, *The Persecutory Imagination: English Puritanism and the Literature of Religious Despair* (Oxford, 1991); Max Weber, *The Protestant Ethic and the Spirit of Capitalism*, trans. Talcott Parsons (Guildford, 1976, first published 1930), 95–139; Jean Delumeau, *Sin and Fear: The Emergence of a Western Guilt Culture, 13th–18th Centuries* (New York, 1990).

66 See note 2 above.

67 James Fisher, The wise virgin, or, a wonderfull narration of the hand of God… in afflicting a childe of eleven years of age [Martha Hatfield] (London, 1653).

